# Financial Implications of GI Bleeding in Patients with LVAD: An Analysis from the US National Inpatient Sample Trends

**DOI:** 10.3390/medsci14010096

**Published:** 2026-02-16

**Authors:** Sudhakar Basetty, Anil Mathew Philip, Roop Sunil Reddy Parlapalli, Naga Sumanth Reddy Gopireddy, Nandakishore Akula, Kalpana Yeddula, Sriveer Kaasam, Lina James George, Revati Varma, Hans Mautong, Kevin John, Ajay Mishra

**Affiliations:** 1 Department of Hospital Medicine, Aspirus Wisconsin Rapids Hospital, Wisconsin Rapids, WI 54494, USA; 2 Department of Internal Medicine, John H Stroger Hospital of Cook County Health, Chicago, IL 60612, USA; anilmathewp@gmail.com (A.M.P.); jameslinaind@gmail.com (L.J.G.); hans.mautong@cookcountyhealth.org (H.M.); 3 Department of Internal Medicine, Geisinger Community Medical Center, Scranton, PA 18510, USA; roopsunil@gmail.com; 4 Division of Nephrology, University of Iowa Hospital, Iowa City, IA 52242, USA; nephro.gopireddy@gmail.com; 5 Department of Internal Medicine, Sanford USD Medical Center, Sioux Falls, SD 57105, USA; nandakishoreakula@gmail.com; 6 Department of Internal Medicine, Aurora Medical Center, Grafton, WI 53024, USA; kalpana.sy@gmail.com; 7 Department of Hospital Medicine, Christus Spohn Hospital—Shoreline, Corpus Christi, TX 78404, USA; sriveerkaasam@gmail.com; 8 School of Health, Universidad Espíritu Santo-Ecuador, Samborondón, Guayaquil 60612, Ecuador; 9 Department of Cardiology, Emory School of Medicine, Atlanta, GA 30322, USA; kevinjohn25@gmail.com; 10 Department of Cardiology, Concord Hospital, Concord, NH 03301, USA; ajaybalasore@gmail.com

**Keywords:** finance, LVAD, GI bleed

## Abstract

**Background**: Gastrointestinal bleeding (GIB) is a common and serious complication in patients with left ventricular assist devices (LVADs), contributing to significant morbidity, prolonged hospitalization, and increased healthcare costs. We evaluated national trends, demographic disparities, and outcomes of GIB in hospitalized LVAD patients. **Methods**: We analyzed adult (≥18 years) LVAD hospitalizations in the National Inpatient Sample (2016–2021), identifying internal LVADs using ICD-10-PCS code 02HA0QZ. GIB was defined using ICD-10-CM codes and classified into upper (UGIB) and lower (LGIB) sources. Survey-weighted logistic and linear regression models assessed associations with mortality, length of stay (LOS), and total charges. Subgroup analyses explored sex and racial disparities. **Results**: Among 20,785 weighted adult LVAD admissions, 9.8% had GIB. Of these, 72.3% had LGIB and 31.0% had UGIB. Patients with GIB were older (59.2 vs. 54.8 years) and more likely to be female (43% vs. 40%) and Black (9.2% vs. 7.8%). GIB was associated with longer LOS (+15.3 days, 95% CI: 12.0–18.5), higher charges (+$316,031, 95% CI: $212,435–$419,627), and greater in-hospital mortality (OR 1.69, 95% CI: 1.25–2.29; *p* < 0.001). Female patients with GIB had higher odds of mortality (OR 1.37) and increased LOS (+5.6 days), though this was not statistically significant. Racial disparities were evident: Black patients with GIB had longer LOS (+8.9 days), while Asian/Pacific Islander patients had shorter LOS (–23.3 days, *p* < 0.001). From 2016 to 2021, GIB prevalence rose modestly (from 9.4% to 10.7%, *p* = 0.33), with no significant change in mortality trends (*p* = 0.13). **Conclusions**: GIB complicates nearly 1 in 10 LVAD hospitalizations, with lower GI bleeds being most common. GIB is independently associated with higher mortality, LOS, and costs. Persistent gender and racial disparities highlight the need for targeted strategies to improve outcomes in this high-risk population.

## 1. Introduction

Left Ventricular Assist Devices (LVADs) are being increasingly used as both a bridge to transplant and as a destination therapy for advanced heart failure. First used experimentally in the late 1960s, LVADs have become more portable and have evolved from pulsatile to continuous-flow devices [1]. In the United States, the annual rate of LVAD placement tripled between 2008 and 2016 [2].

While LVADs improve survival and quality of life, they are also linked to gastrointestinal bleeding (GIB), a common and clinically significant complication with an incidence of up to 30% [3]. GIB in LVAD patients has multiple causes, including acquired von Willebrand factor (vWF) deficiency, altered shear stress, anticoagulation therapy, and arteriovenous malformation (AVM) formation [4]. Blood interaction with the LVAD leads to acquired von Willebrand disease; shear forces cause proteolysis of vWF multimers and impair platelet adhesion [5,6]. Reduced arterial pulsatility leads to mucosal hypoperfusion and regional hypoxia, which promote arteriovenous dilation and the formation of angiodysplasia [7]. Cardiac transplantation reverses these changes and normalizes vWF levels, as reported by Uriel et al. [8]. The use of antiplatelet and anticoagulation therapy further increases bleeding risk in these patients [3].

Although previous studies have explored clinical outcomes in LVAD patients with GIB, few have assessed demographic factors, insurance status, socioeconomic status, complications, and healthcare costs. This study examines healthcare costs and complications, including acute coronary syndrome, cardiac arrhythmias, pulmonary embolism, stroke, and acute kidney injury, across different socioeconomic groups with insurance disparities. Using NIS data from 2016 to 2021, we will evaluate demographic characteristics, morbidity, inpatient outcomes, length of stay, economic impact, and complications in LVAD patients with GIB.

## 2. Materials and Methods

Study design and data origin:

The data for this retrospective study were taken from the National Inpatient Sample (NIS), a publicly available database that includes all-payer inpatient data from the United States. NIS was developed under the Healthcare Cost and Utilization Project (HCUP) by the U.S. Agency for Healthcare Research and Quality (AHRQ). We collected data on 20,785 patients aged ≥18 years from NIS 2016–2021, identifying inpatients with LVADs and GI bleed using the ICD-10-PCS code 02HA0QZ and ICD-10-CM codes, respectively.

Study Population:

Patients aged 18 years or older admitted with a left ventricular assist device (LVAD) were included. Among these, cases with gastrointestinal bleeding (GIB) were identified using the ICD-10-CM diagnosis code.

Population variables:

Study population characteristics included age, sex, and race. Additional variables were socioeconomic status (SES) and primary payer. SES was determined using median household income quartiles for each patient’s ZIP code, as provided in the NIS. The payer source was categorized as Medicare, Medicaid, private insurance, or self-pay/other.

Hospital-based variables:

Hospital variables included bed size (small, medium, or large), location (rural or urban), and teaching status. These factors were used to assess financial implications, resource utilization, and healthcare delivery among LVAD patients with GIB.

Outcomes:

The primary outcome was inpatient hospital mortality and financial burden among LVAD patients with GIB, whereas the secondary outcome included length of hospitalization, insurance distribution, and complications (cardiac arrythmias, heart failure, acute coronary syndrome, pulmonary embolism, and ischemic/hemorrhagic stroke).

Statistical analysis:

All statistical analyses were conducted using Stata^®^ Version 18.5 (StataCorp, College Station, TX, USA). A two-sided *p* < 0.05 was considered statistically significant. Binary outcomes for primary and secondary endpoints were analyzed using survey-weighted logistic regression, while continuous outcomes (charges and length of stay) were analyzed using linear regression. Confounding variables, including patient demographics, comorbidities, insurance payer type, hospital size, and socioeconomic status, were adjusted using multivariate regression analysis. A *p*-value of less than 0.05 was considered statistically significant.

Baseline patient and hospital characteristics, as well as incidence estimates, including the distribution of gastrointestinal bleeding, were derived from survey-weighted descriptive analyses and are unadjusted. These results describe the study population and underlying distributions.

Analyses of associations between gastrointestinal bleeding and clinical outcomes used multivariable, survey-weighted regression models and were adjusted. Specifically, outcomes such as length of stay, total hospital charges, in-hospital mortality, and cardiovascular and thromboembolic complications were evaluated with multivariable linear or logistic regression, as appropriate. These models adjusted for patient and hospital covariates, including demographics, socioeconomic status, and hospital characteristics.

Unadjusted results cover descriptive characteristics. In contrast, all outcome associations presented in the results section reflect adjusted estimates. 

Limitations:

This study has important limitations. First, this study reports healthcare charges rather than actual costs, as the NIS does not provide cost data. Second, the absence of key clinical variables in the NIS, including LVAD type, anticoagulation intensity, and bleeding severity, limits the ability to draw definitive mechanistic or causal conclusions. Third, as an observational, cross-sectional study, causal relationships cannot be established. Accordingly, race, ethnicity, and sex-based differences are presented as exploratory and hypothesis-generating, particularly in smaller subgroups. Finally, administrative data analyses may be subject to misclassification bias and limit clinical granularity.

## 3. Results

Of 20,785 adult LVAD hospitalizations, 2045 (9.8%) developed gastrointestinal bleeding (GIB). Lower GI bleeding was more common, accounting for 74.8% of cases, while upper GI bleeding comprised 30.7%.

**Figure 1:**
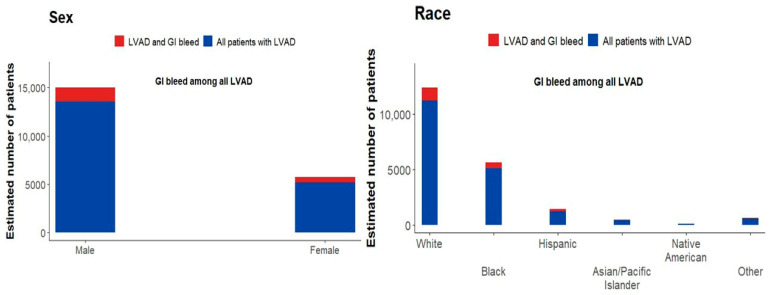
Unadjusted variables: sex, race, bed size, and hospital location.

Table 1 summarizes baseline demographics of LVAD patients, stratified by gastrointestinal bleeding. The mean age was 56.7 years (95% CI 56.3–57.2) for all LVAD patients and 59.2 years (95% CI 58.0–60.4) for those with GIB. Both groups were predominantly male (72.3% in LVAD, 71.8% in LVAD + GIB). White patients were the largest group (59.6% in LVAD, 57% in LVAD + GIB), followed by black (27.3% and 26.8%) and Hispanic patients (7% and 9.2%). Smaller proportions were Asian/Pacific Islander, Native American, or Other as shown in Figure 1. Patients with GIB were slightly older and more frequently Hispanic, while sex and other racial distributions were similar.

**Figure 2:**
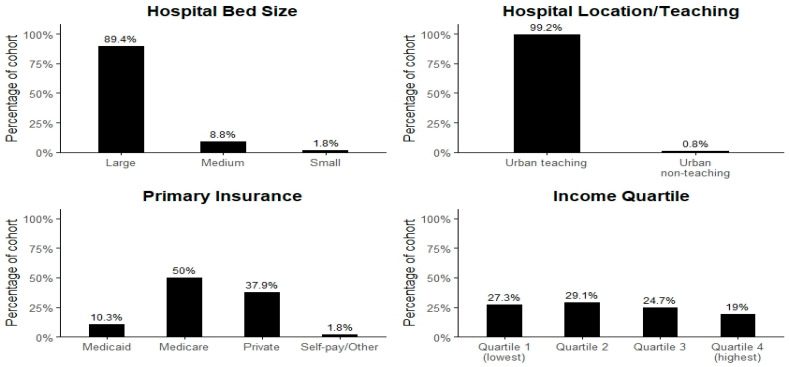
Unadjusted variables: percent distribution by hospital bed size, location, and socioeconomic status.

Among LVAD patients with gastrointestinal bleeding, Medicare was the predominant payer (50%), followed by private insurance (38%), with smaller proportions on Medicaid (10%) or self-pay/other (2%). Patients were more frequent from lower- and middle-income ZIP codes (56% in quartiles 1, 2, and 3), reflecting socioeconomic vulnerability in this cohort. Nearly all LVAD + GIB admissions occurred in large, urban teaching hospitals (≈89% and 99%, respectively), underscoring the concentration of advanced device care in high-resource centers as shown in Figure 2.

In terms of resource use, baseline LVAD admissions averaged $400,427, while the occurrence of GIB added approximately $337,000 in incremental charges, increasing the mean hospitalization cost to $737,567. These findings highlight the substantial economic burden associated with GIB in LVAD patients, layered on top of already high baseline hospitalization costs, shown in Table 2.

Among LVAD patients, gastrointestinal bleeding was associated with a significantly increased risk of complications. The odds of developing acute coronary syndrome were about 1.5-fold higher, while the risk of acute kidney injury was nearly 1.6-fold higher compared with LVAD patients without GIB. In contrast, no significant associations were found for ischemic stroke, hemorrhagic stroke, cardiac arrhythmias, heart failure, or pulmonary embolism, suggesting that the excess morbidity in this population is driven primarily by cardiovascular ischemic events and renal injury.

Complications following GIB in LVAD patients did not differ significantly by sex; for example, female sex was not associated with ACS (OR 1.09, 95% CI 0.81–1.45, *p* = 0.57) or ischemic stroke (OR 0.77, 95% CI 0.52–1.15, *p* = 0.20). By race, however, significant differences emerged. Black patients had a markedly lower risk of ACS compared to whites (OR 0.29, 95% CI 0.19–0.44, *p* < 0.001), while Asian/Pacific Islander patients had substantially higher odds of hemorrhagic stroke (OR 3.78, 95% CI 1.25–11.39, *p* = 0.018). No other racial groups showed statistically significant associations for complications as shown in Table 3.

**Figure 3:**
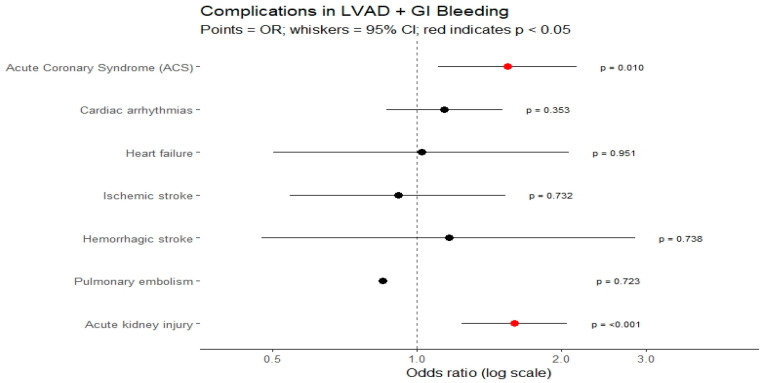
Adjusted complications in LVAD + GI bleed.

In the overall LVAD population, gastrointestinal bleeding was associated with a 1.69-fold increase in the odds of in-hospital mortality (OR 1.69, 95% CI 1.25–2.29, *p* < 0.001) (Figure 3). Between 2016 and 2021, the incidence of GI bleeding among LVAD patients increased gradually. In contrast, mortality among LVAD patients with GI bleeding did not change significantly over time (*p* = 0.13).

## 4. Discussion

LVADs are increasingly used as life-sustaining therapy for patients with advanced or end-stage heart failure. In the United States, over 20,000 LVADs are implanted annually in more than 180 hospitals. These devices serve as a bridge to transplantation or as destination therapy for non-transplant candidates. Gastrointestinal bleeding (GIB) is the most common complication, resulting from mechanisms such as AVM, acquired vWF deficiency, coagulopathy, and anticoagulation therapy. Previous studies report a GIB incidence of 15% to 25% in LVAD patients [4,9]. A systematic review found a pooled GIB prevalence of 23% (95% CI, 20.5–27%), with upper GIB at 48% (95% CI, 39–57%) and lower GIB at 22% (95% CI, 16–31%) [10]. While prior studies found upper GIB more common, our findings show a predominance of lower GIB (72.3%) over upper GIB (31%). Possible contributing factors include patient characteristics (age, comorbidities such as prior colonic disease or diverticulosis), diagnostic bias (timing of endoscopy), and institutional protocols (use of prophylaxis for upper GIB).

In our analysis of LVAD patients from NIS 2016–2021, those who developed GIB were older than the overall LVAD population (59.2 years vs. 56.7 years), indicating that age is a significant risk factor for bleeding events. This aligns with previous studies showing that advanced age is associated with fragile blood vessels, higher rates of angiodysplasia, and increased exposure to anticoagulation therapy, all contributing to elevated GI bleeding risk in older LVAD recipients [11].

Sex distribution was similar between groups, with a predominance of male patients. This is consistent with previous studies indicating that men are more likely to undergo LVAD placement due to epidemiologic and clinical factors influencing advanced heart failure management [12].

White patients made up the majority of both LVAD and LVAD + GIB groups, consistent with national trends. However, a slightly higher proportion of Hispanic patients developed GI bleeding. This finding highlights potential racial and ethnic disparities in access to care, underlying comorbidities, or post-implantation management.

Most hospitalizations were covered by Medicare (50%), followed by private insurance (38%), with Medicaid (10%) and self-pay/other (2%) accounting for fewer cases. The predominance of Medicare reflects age-related eligibility among advanced heart failure patients. Low Medicaid representation highlights persistent access barriers for younger patients, including delayed diagnosis, referral, and insurance preauthorization. These findings underscore healthcare access inequities and system-level challenges in the referral process [13]. Our results are consistent with previous research showing that Medicare and privately insured patients are more likely to receive advanced heart failure interventions than those with Medicaid [14].

Urban teaching hospitals accounted for approximately 89% of all LVAD and nearly 99% of LVAD-associated GIB admissions. Our study results demonstrate that most LVAD patients with GIB were treated at large, urban, teaching hospitals, reflecting the concentration of advanced medical support, specialized equipment, and multiple specialties, as well as improved data reporting to national registries such as NIS and INTERMACAS (Interagency Registry for Mechanical Assisted Circulatory Support) [15].

Most LVAD patients with GIB were from the lowest to middle-income zip codes (Quartiles 1, 2, and 3). This reflects socioeconomic vulnerability in the cohort, as evidenced by higher complication rates, delayed access to outpatient care, greater comorbidity burden, longer hospitalizations, and a higher financial burden. Overall, these factors magnify the economic burden, which reinforces the financial focus of the study.

The average hospitalization cost for baseline LVAD admissions was approximately $400,427, with GI bleeding increasing the cost by nearly $337,000, resulting in a total mean cost of $737,567. These findings underscore the significant economic impact of LVAD-related complications. The high baseline cost reflects the procedure’s complexity and resource demands. Our results are consistent with previous studies showing higher costs for LVAD admissions complicated by GI bleeding. A study conducted in 2009–2020 using the NIS dataset demonstrates that the median hospitalization cost was higher in LVAD patients with GIB ($275,696.23) than in non-GIB patients ($203,515.67) [16,17]. The increased costs are likely due to additional diagnostic procedures, specialty consultations, blood transfusions, treatment, and longer hospital stays [18].

Our study found that LVAD patients with GIB had 1.6-fold higher odds of developing acute kidney injury (AKI) compared to those without GIB. This aligns with the previous literature reporting pooled AKI incidence rates of 37% and severe AKI requiring RRT at 13% in LVAD patients. The pathophysiology is multifactorial [19,20,21], including mechanical stress-induced hemolysis, acquired von Willebrand disease, and decreased blood volume from GI bleeding via AVMs [19]. LVAD patients who develop AKI also have higher odds of 30-day and 1-year mortality [22].

LVAD patients with GIB had 1.5-fold higher odds of developing acute coronary syndrome (ACS) compared to those without GI bleed. Temporarily holding anticoagulation to manage GIB increases the risk of pump thrombosis, which can lead to ACS. Hemodynamic instability from GI bleeding may also contribute to ACS, even in the absence of thrombus formation [23].

Other complications among LVAD patients with GIB included heart failure, pulmonary embolism, cardiac arrhythmias, and ischemic or hemorrhagic stroke, with no statistically significant differences in rates. By race, black patients had lower odds of developing ACS compared to whites (OR 0.29, 95% CI 0.19–0.44, *p* < 0.001), and Asian/Pacific Islander patients had higher odds of hemorrhagic stroke (OR 3.78, 95% CI 1.25–11.39, *p* = 0.018). Lower ACS rates in black patients may be due to a smaller patient population. Further studies are needed to clarify the risk factors [11,24]. Overall, a higher percentage of complications was observed in the advanced age group, aligning with Medicare payer data and correlating with increased length of stay, higher costs, and greater post-acute care needs [25,26].

In our analysis of 20,785 LVAD inpatients, 9.8% experienced GIB, with most cases involving lower GI bleeding. GIB was associated with a significantly longer hospital stay (average 15 days; 95% CI 12.0–18.5) and higher hospital mortality (OR 1.69, 95% CI 1.25–2.29, *p* < 0.001) compared to LVAD patients without GI bleed. These results highlight the substantial clinical and healthcare burden of GIB in LVAD patients. Black patients with GIB had longer hospital stays, consistent with previous studies [25]. There is no prior published data on shorter stays among Asian/Pacific Islander LVAD patients with GI bleed, making this a potentially unique finding. Contributing factors may include patient comorbidities, disease severity, age, hospital resources, multidisciplinary care, early intervention, and social support. Further studies with larger Asian/Pacific Islander samples and analysis of mediators are needed to better understand length of stay.

From 2006 to 2021, GIB prevalence increased slightly from 9.4% to 10.7% (*p* = 0.33), but mortality among LVAD patients with GIB did not change significantly (coefficient per year = –0.12, *p* = 0.13). Stable mortality rates may reflect advances in device technology, improved anticoagulation protocols, and better management in specialized centers [26,27].

Our results align with previous large-scale studies that show the significant impact of GIB on patient outcomes. A study using NIS 2009–2020 found higher odds of mortality (OR 2.801, 95% CI 2.216–3.539) and longer hospital stays (median 44 days vs. 29 days) in LVAD patients with GIB [17]. Another nationwide study (NIS 2008–2017) reported a 2.53-day increase in length of stay (95% CI 1.78–2.98; *p* < 0.001) in LVAD patients with GIB, with no significant difference in mortality [12]. In contrast, our analysis shows a greater increase in length of stay (+15 days) and a statistically significant increase in hospital mortality (*p* < 0.001).

## 5. Conclusions

LVAD implantation in patients with advanced heart failure offers a survival advantage and improves quality of life. However, GIB is significantly more prevalent and remains the most common complication among LVAD recipients. In this cohort, GIB was predominantly observed in older patients from lower-income ZIP codes and among Medicare beneficiaries. This disproportionate impact on a vulnerable population imposes a significant financial burden on the healthcare system. To address these clinical complications and rising medical costs, greater emphasis should be placed on prevention strategies, multidisciplinary care, comprehensive risk assessment, and patient-specific therapeutic approaches.

## Figures and Tables

**Table 1:** Baseline demographics of LVAD patients with and without gastrointestinal bleeding (unadjusted).

Characteristics	All LVAD (N = 20,785)	LVAD + GIB (N = 2045)
Mean Age, years (95% CI)	56.7(56.3–57.2)	59.2(58.0–60.4)
Sex (%)		
Male	72.3	71.8
Female	27.7	28.2
Race (%)		
White	59.6	57.0
Black	27.3	26.8
Hispanic	7.0	9.2
Asian/Pacific Islander	2.3	2.1
Native American	0.7	0.8
Other	3.2	4.2

**Table 2:** Unadjusted variables: insurance, hospital characteristics, and total charges in LVAD patients with gastrointestinal bleeding.

Characteristic	LVAD + GIB (N = 2045)
Primary Insurance (%)	
Medicare	50.0
Medicaid	10.3
Private	37.9
Self-pay/Other	1.8
ZIP-code Income Quartile (%)	
Quartile 1 (lowest)	27.3
Quartile 2	29.1
Quartile 3	24.7
Quartile 4 (highest)	19.0
Hospital Bed Size (%)	
Small	1.8
Medium	8.8
Large	89.4
Hospital Location/Teaching (%)	
Urban non-teaching	0.8
Urban Teaching	99.2
Mean Total Charges ($)	$737,567 [$400,427(baseline) + $337,140; 95% CI $632,322–$842,957]

**Table 3:** Adjusted complications in LVAD and gastrointestinal bleeding.

Complication	Odds Ratio	95% CI	*p*-Value
Acute Coronary Syndrome (ACS)	1.546	1.110–2.154	0.010
Cardiac arrhythmias	1.141	0.864–1.509	0.353
Heart failure	1.022	0.504–2.076	0.951
Ischemic Stroke	0.914	0.545–1.532	0.732
Hemorrhagic stroke	1.165	0.476–2.853	0.738
Pulmonary embolism	0.848	0.341–2.110	0.723
Acute kidney injury	1.598	1.242–2.058	<0.001

## Data Availability

The original data presented in this study are openly available in the National Inpatient Sample at National (Nationwide) Inpatient Sample (NIS).

## Abbreviations

The following abbreviations are used in this manuscript:

GIB	Gastrointestinal Bleeding
LVAD	Left Ventricular Assist Device
US	United States
ICD	International Classification of Diseases
UGIB	Upper GI Bleeding
LGIB	Lower GI Bleeding
LOS	Length of Stay
CI	Confidence Interval
OR	Odds Ratio
vWF	von Willebrand factor
NIS	National Inpatient Sample
